# Supplementary figures and images for: Ursolic acid regulates gut microbiota and corrects the imbalance of Th17/Treg cells in T1DM rats
Source: PLoS One. 2022 Nov 3;17(11):e0277061. doi: 10.1371/journal.pone.0277061 (PMC9632920; doi:10.1371/journal.pone.0277061)

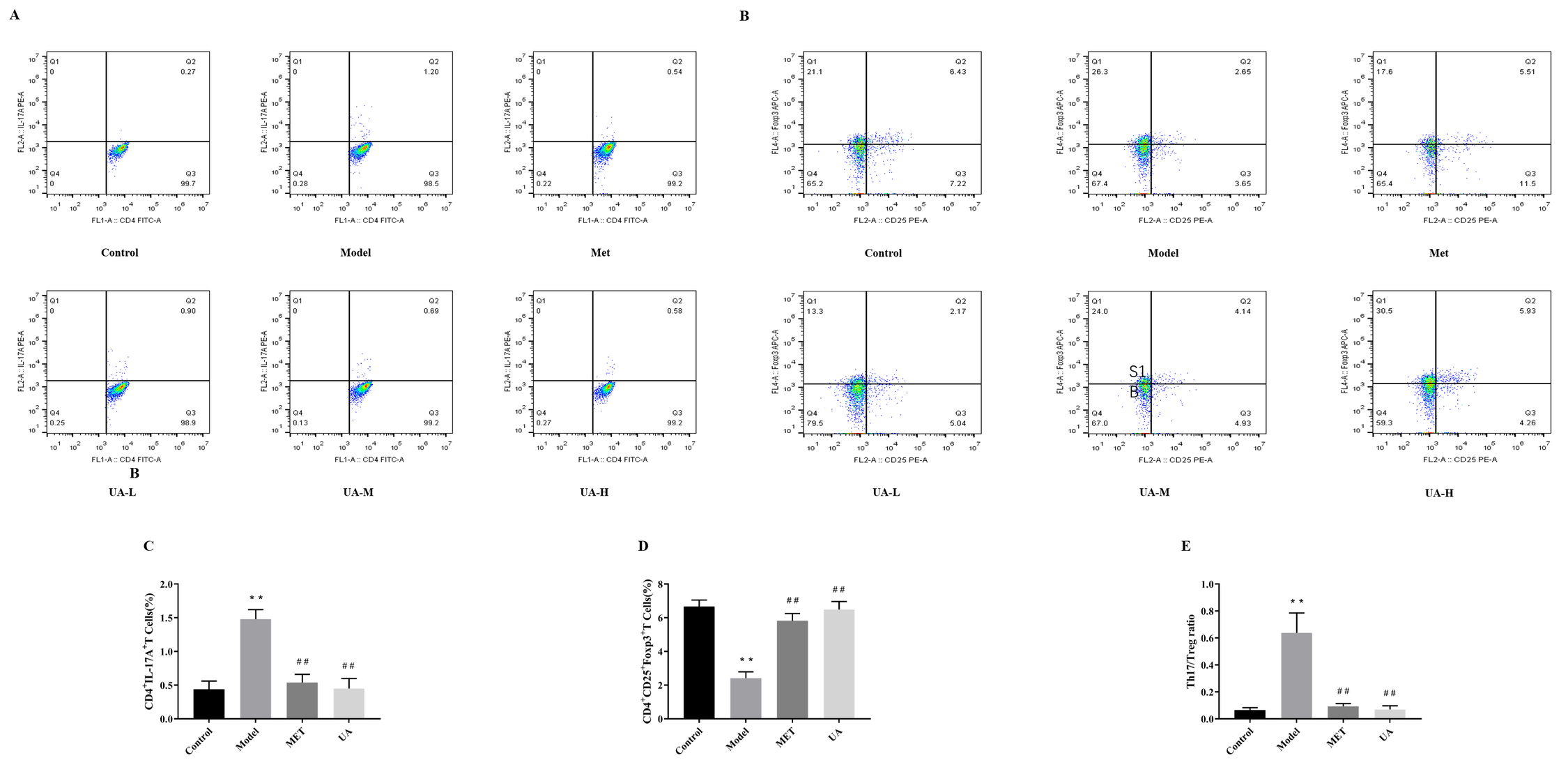

Supplement: S1 Fig — (A) CD4+IL-17A+ (Th17) cells and (B) CD4+CD25+Foxp3+ (Treg) cells in mesenteric lymph nodes from the six groups were analyzed by flow cytometry. (C) The percentage of CD4+IL-17A+(Th17) cells in mesenteric lymph nodes. (D) The percentage of CD4+CD25+Foxp3+ (Treg) cells in mesenteric lymph nodes. (E) The Th17/Treg ratio in mesenteric lymph nodes. (n = 6). The data are expressed as the mean ± SD. **P<0.01 versus the control, *P<0.05 versus the control; ##P<0.01 versus Model, #P<0.05 versus Model. (TIF) [file pone.0277061.s002.tif]
