# Supplementary material for: Ursolic acid regulates gut microbiota and corrects the imbalance of Th17/Treg cells in T1DM rats
Source: PLoS One. 2022 Nov 3;17(11):e0277061. doi: 10.1371/journal.pone.0277061 (PMC9632920; doi:10.1371/journal.pone.0277061)
Supplement: S1 Raw images — (PDF) [file pone.0277061.s003.pdf]

Repeat 1

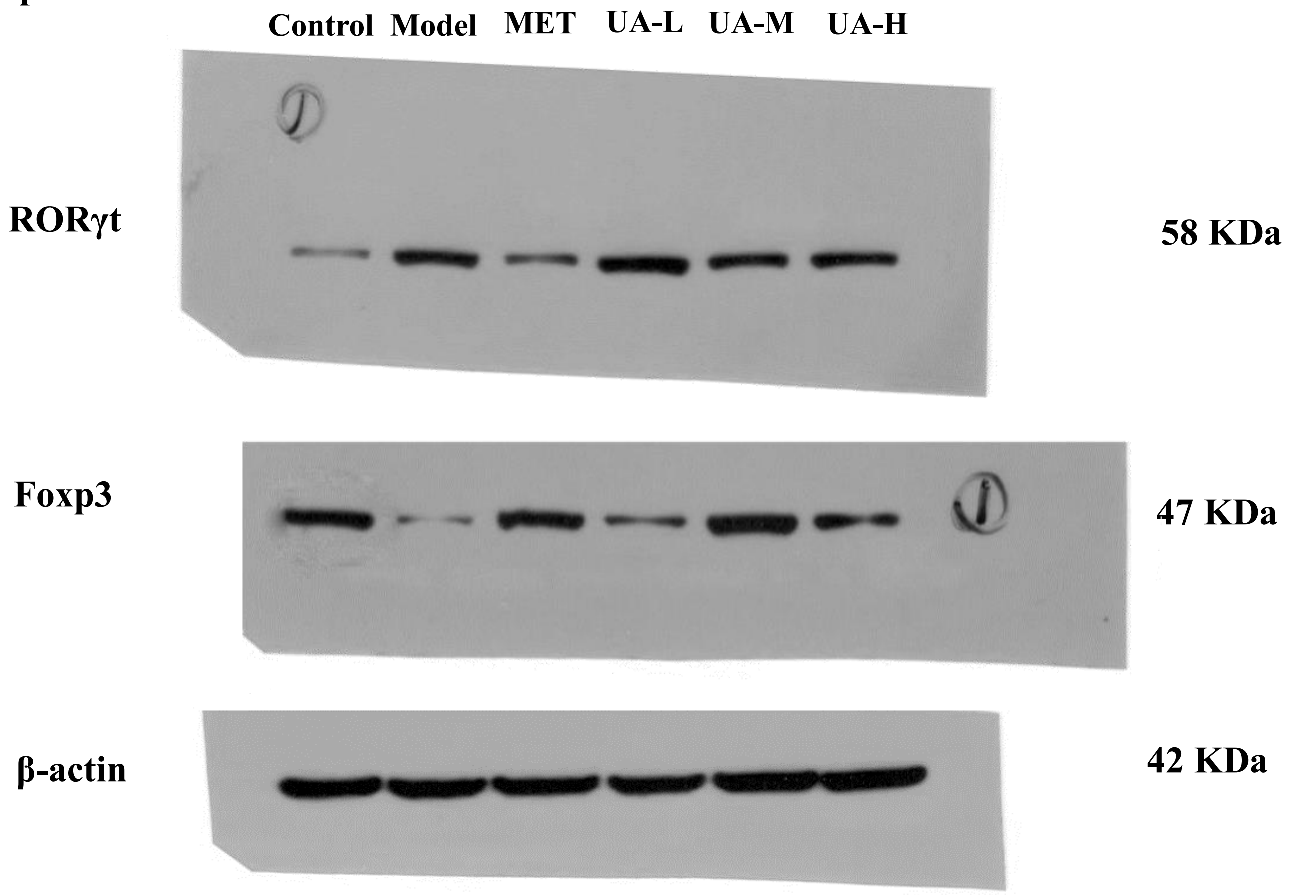

Repeat 2

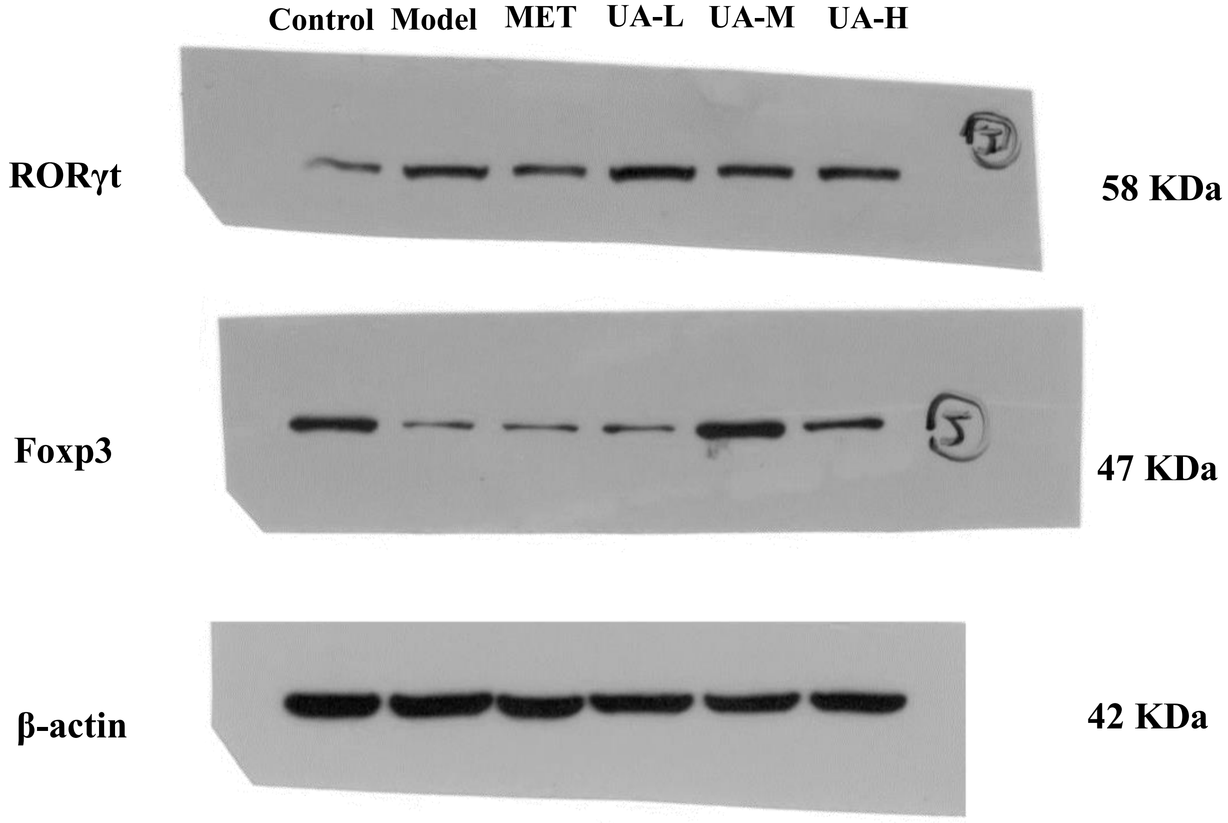

Repeat 3

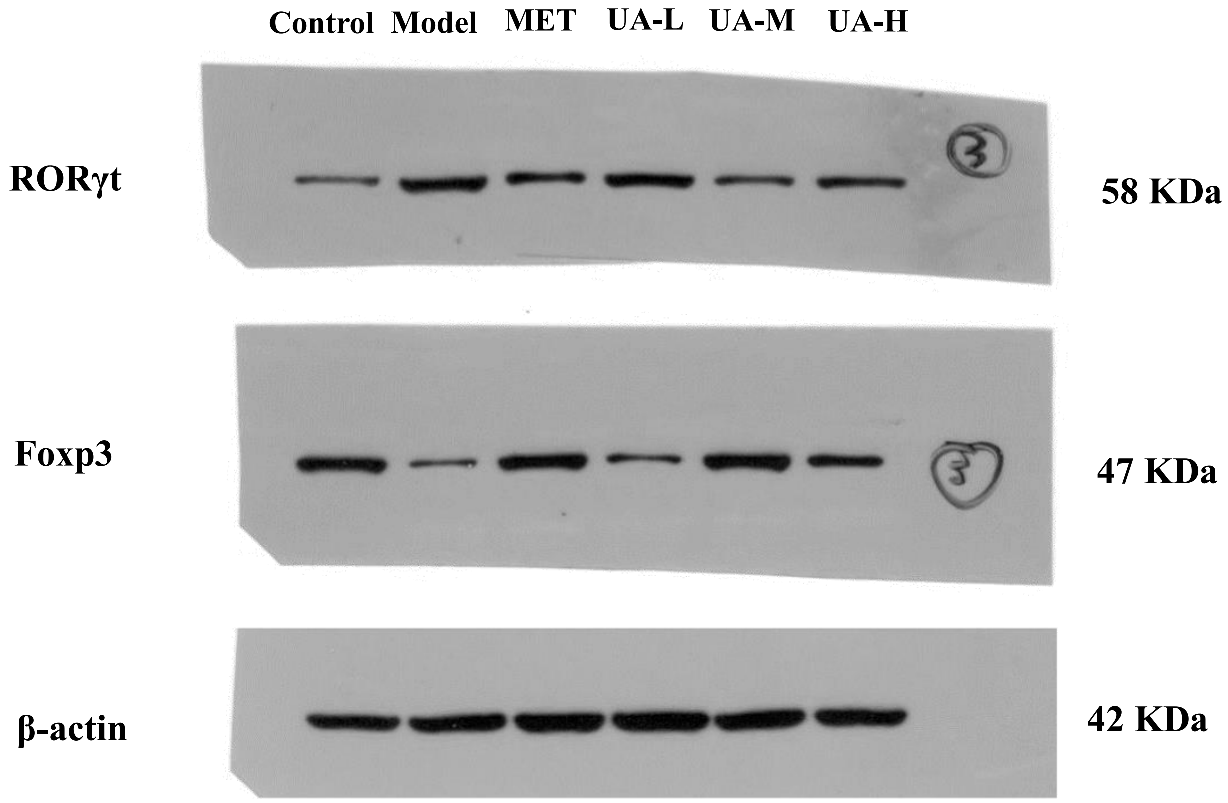
Control
Model
MET
UA-L
UA-M
UA-H
RORγt
58 KDa
Foxp3
47 KDa
β-actin
42 KDa

Groups	The gray value of the band			Gray value ratio between the target band and the internal reference band		mean value	
	**Foxp3**	**RORγt**	**β-Actin**	**Foxp3**	**RORγt**	**Foxp3**	**RORγt**
**Normal**	176904	39125	300960	0.59	0.13	0.59	0.16
	173026	47189	314592	0.55	0.15		
	152269	49963	237920	0.64	0.21		
**Model**	45401.6	147555	283760	0.16	0.52	0.19	0.58
	61110	164997	305550	0.20	0.54		
	49295	159623	234740	0.21	0.68		
**MET**	157172	78586	291060	0.54	0.27	0.55	0.33
	140352	99416	292400	0.48	0.34		
	149730	91770	241500	0.62	0.38		
**UA-L**	92996	139495	290614	0.32	0.48	0.32	0.47
	82656	128577	306135	0.27	0.42		
	94354	124150	248300	0.38	0.50		
**UA-M**	197644	108385	318780	0.62	0.34	0.67	0.32
	186845	91940	296580	0.63	0.31		
	186525	73628	245427	0.76	0.30		
**UA-H**	111794	133532	310540	0.36	0.43	0.41	0.40
	125649	105195	292208	0.43	0.36		
	107042	97528	237872	0.45	0.41		
