# Supplementary material for: Ursolic acid regulates gut microbiota and corrects the imbalance of Th17/Treg cells in T1DM rats
Source: PLoS One. 2022 Nov 3;17(11):e0277061. doi: 10.1371/journal.pone.0277061 (PMC9632920; doi:10.1371/journal.pone.0277061)
Supplement: Attachment — Submitted filename: Response to Reviewers.docx [file pone.0277061.s004.docx]

Dear Editors and Reviewers:

Thank you very much for giving us an opportunity to revise our manuscript, we appreciate the reviewers very much for their positive and constructive comments and suggestions on our manuscript entitled “Ursolic acid regulates gut microbiota and corrects the imbalance of Th17/Treg cells in T1DM rats” (Manuscript Number: PONE-D-22-16987) by Weiwei Chen et al submitted to PLOS ONE. Those comments are all valuable and very helpful for revising and improving our paper, as well as the important guiding significance to our researches. Appended to this letter is our point-by-point response to the comments raised by the reviewers. The comments are reproduced and our responses are given directly afterward in a different color (blue). And we wish it to be reconsidered for publication in PLOS ONE. A list of changes and responses to the reviewers are as follows:

**Answer Editor-in-Chief:**

*1. It lacks conclusive and mechanistic experiments.*

**Reply:** We gratefully appreciate for your comment. Indeed, our study will be more profound if we get the relevant results in conclusive and mechanistic experiments. This study focuses on the protective effect and potential mechanism of ursolic acid (UA), a natural pentacyclic triterpenoid extracted from fruit and a number of traditional Chinese medicinal plants, on type 1 diabetes mellitus (T1DM) in rats. We found that UA treatment significantly alleviated the progressive destruction of pancreatic β-cells induced by streptozotocin (STZ); rescued weight and fasting blood glucose (FBG) levels; regulated the expression levels of cytokines (IL-17A, IL-10 and TNF-α) and transcription factors (RORγt and Foxp3). Importantly, our study results demonstrated that UA treatment increases the diversity and uniformity of gut microbiota in T1DM rats, as well restores the Treg/Th17 cell balance. However, the correlation between the protective effect of UA on T1DM and the effect of UA in regulating gut microbiota and correcting Th17/Treg balance has not been adequately discussed and validated, which is what we're going to do next. Considering the complexity of the body and the biological diversity of the gut microbiota, we hope to use germ free rats to carry out related research in the next experiment. AS the results of experimental cannot be obtained in a short period of time, they cannot be shown in this article. Therefore, we seek for the editor’s tolerance and understanding. And we have revised the manuscript to downplay this claim by revising the writing of the Abstract, Results and Discussion/Conclusion sections as suggested by the reviewer. For example, we mentioned that protective effects of UA against T1DM are being explored in abstract, and these findings suggest that UA can lower FBG levels in T1DM rats, delay the progressive destruction of pancreatic β-cells, and modulate gut microbiota homeostasis and immune function in rat with streptozotocin-induced T1DM. In discussion, we removed the hypothesized that ursolic acid alleviates the symptoms of T1DM by acting on the gut microbiota. In the conclusion, we also replaced the statement that UA might relieve symptoms by regulating gut microbiota and correcting the differentiation of Th17/Treg cells in T1DM rats into our study demonstrated that UA alleviates the symptoms of STZ-induced T1DM in rat. UA treatment reduced FBG levels, delayed the progressive destruction of pancreatic β-cells, modulated intestinal microflora composition, and restored Treg/Th17 cell balance.

*2. It was also felt that the authors need to discuss the rationale for use of ursolic acid and how it compares to metformin.*

**Reply:** We thank you for reminding us this important point. According to your suggestion, we have detailed the mechanism in the manuscript in Page 15-16 line 341-360 as shown below:

T1DM is characterized by the immune-mediated depletion of β-cells, resultanting in insulin deficiency and hyperglycaemia. Metformin could decrease glucose levels [28-30] and improves insulin sensitivity [31]. The American Diabetes Association has also made a positive recommendation for adding metformin to insulin therapy in overweight patients with T1DM [32]. Therefore, metformin was used as a positive control in our study.

UA is a natural pentacyclic triterpenoid derived from fruit and various traditional Chinese medicinal plants. It also possesses important antidiabetic effects, including lowering blood glucose levels and preserving pancreatic islet cells function [27, 33, 34]. For instance, UA exerts hypoglycemic effects by inhibiting the activities of α-amylase and α-glucosidase [35]; activating TGR5 to enhance GLP-1secretion in type 1-like diabetic rats [36]; and reducing the activity of protein tyrosine phosphatase 1 B [37].Compared to metformin, UA has been shown to exhibit poor oral bioavailability, low solubility, and intestinal permeability [38]. However, metformin is associated with rare but serious events of lactic acidosis and gastrointestinal side effects [39, 40], yet UA did not exhibit any significant toxicity in human THP-1 cell [41]. Moreover, a long-term toxicity study showed that oral dosing of UA for 90 consecutive days did not lead to toxic effects at any of the doses. In contrast, recent studies have reported that gut microbiota dysbiosis and the differentiation of Th17/Treg cells contribute to T1DM progression [11, 17, 42]. In addition, UA modulates the abundance and diversity of intestinal flora and inhibit Th17 cells differentiation [43, 44]. However, whether UA has similar protective and ameliorative effects on T1DM remains unknown.

*3. Many other important concerns including the language needs to be addressed by a point by point response to all the comments of the reviewers*

**Reply:** Thank you for your significant reminding. All article has been edited by professionals, and the edit certificate has been uploaded. Revised portion is marked in red on the paper to mark all changes clearly. A screenshot of the retouching certificate as follows:

Other important concerns will be addressed by a point by point response to all the comments of the reviewers.

**Response to Journal Requirements:**

*1. Please ensure that your manuscript meets PLOS ONE's style requirements, including those for file naming.*

**Reply:** Thank you for reminding us this important point. All article has been edited by professionals, and we have retyped our manuscript to meets PLOS ONE's style requirements.

*2.As part of your revision, please complete and submit a copy of the Full ARRIVE 2.0 Guidelines checklist, a document that aims to improve experimental reporting and reproducibility of animal studies for purposes of post-publication data analysis and reproducibility.*

**Reply:** Thank you for your significant reminding. A copy of the Full ARRIVE 2.0 Guidelines checklist has been completed as requested and will be submitted as supporting information.

*3. We note that you have stated that you will provide repository information for your data at acceptance. Should your manuscript be accepted for publication, we will hold it until you provide the relevant accession numbers or DOIs necessary to access your data. If you wish to make changes to your Data Availability statement, please describe these changes in your cover letter and we will update your Data Availability statement to reflect the information you provide.*

**Reply:** Thank you for reminding us this important point.

*4. PLOS ONE now requires that authors provide the original uncropped and unadjusted images underlying all blot or gel results reported in a submission’s figures or Supporting Information files.*

**Reply:** Thank you for reminding us this important point. We will provide the original uncropped and unadjusted images underlying all blot or gel results reported in Supporting Information files.

*5. PLOS requires an ORCID iD for the corresponding author in Editorial Manager on papers submitted after December 6th, 2016. Please ensure that you have an ORCID iD and that it is validated in Editorial Manager. To do this, go to ‘Update my Information’ (in the upper left-hand corner of the main menu), and click on the Fetch/Validate link next to the ORCID field. This will take you to the ORCID site and allow you to create a new iD or authenticate a pre-existing iD in Editorial Manager.*

**Reply:** Thank you for your significant reminding.

**Responses to reviewers' comments to authors:**

*1. Is the manuscript technically sound, and do the data support the conclusions?
The manuscript must describe a technically sound piece of scientific research with data that supports the conclusions. Experiments must have been conducted rigorously, with appropriate controls, replication, and sample sizes. The conclusions must be drawn appropriately based on the data presented.*

*Reviewer #1: Yes*

*Reviewer #2: Yes*

**Reply:** Thank you very much for your careful reading and recognition of our manuscript.

*2. Has the statistical analysis been performed appropriately and rigorously?*

*Reviewer #1: No*

*Reviewer #2: I Don't Know*

**Reply:** We are very sorry for our careless mistake and Thank you for reminding us this important point. We have examined statistical methods and procedures and reanalyzed the data. Statistical comparisons among multiple groups were analyzed for significance using one-way ANOVA followed by the least significant difference test analysis. And R results are expressed as mean ± standard deviation. P<0.05 was considered statistically significant at p < 0.05. For example, in the Results section, we described the statistical results in text and added the statistical results to Fig 1A and the corresponding legend.

*3. Have the authors made all data underlying the findings in their manuscript fully available?
Reviewer #1: Yes*

*Reviewer #2: Yes*

**Reply:** Thank you very much for your careful reading and recognition of our manuscript.

*4. Is the manuscript presented in an intelligible fashion and written in standard English?
Reviewer #1: Yes*

*Reviewer #2: Yes*

**Reply:** Thank you very much for your careful reading and recognition of our manuscript.

**Answer Reviewer #1:**

**Major concerns**
*1. The figures are difficult to follow, they are not labeled correctly and in some the statistics have not been conducted (Fig 1A, B, Fig 2B, C etc.). Please make sure each figure is in one page with sub figures labeled accordingly. In addition, the figure legends should be very descriptive, and any abbreviation used (eg MET) should be defined in the legend as well.*

**Reply:** Thanks for your valuable counsel. We are so sorry that this part was not clear in the original manuscript, and we have revised the contents of this part. Firstly, we have relabeled all the figures to match the figure file name and the caption label with the corresponding in-text citations in our manuscript. At the same time, we rearranged the figures to put each figure in one page with sub figures labeled. Then, we redescribed the key information of each figure in the corresponding legend to make it easier for the reader to understand. All abbreviations have been defined in the legend as well. Lastly, we have examined statistical methods and procedures and reanalyzed the data. Statistical comparisons among multiple groups were analyzed for significance using one-way ANOVA followed by the least significant difference test analysis. And R results are expressed as mean ± standard deviation. *P*<0.05 was considered statistically significant at p < 0.05. our statistical results were shown in figure files and explained in the legend.

**3.1UA treatment alleviated pancreatic β cells injury and reduced FBG level in T1DM rats.**

To assess the effects of UA on T1DM rats, we monitored the body weight and FBG levels of the rats in all groups. As expected, after intraperitoneal injection of STZ, the body weight of T1DM rats began to significantly decrease, whereas it continued to increase in the control group. Compared with the model group, the body weights of rats in the UA-M, UA-H and MET groups were notably increased after 6 weeks of treatment(*P*<0.01) (Fig 1A). One week after STZ injection, all rats in Model and treatment groups developed a higher FBG level than control group(*P*<0.01).  Treatment with both UA and MET led to a less increase in FBG compared to the T1DM rats(*P*<0.01) (Fig 1B). Notably, histological analysis showed that the model group displayed distinctly abnormal islet structures compared with those of the control group; they appeared as small islets with irregular morphology and indistinct boundaries with surrounding tissue, inhomogeneous islet cells, cytoplasmic vacuolation, fat vacuoles, and infiltration of inflammatory cells into islets. However, UA-treated rats had a significantly larger islet surface area and a lower number of insulin-positive cells in the islets compared to the rats in the model group (Fig 1C). Together, these results indicated that UA treatment significantly ameliorated STZ-induced T1DM.

**Fig 1. UA treatment alleviated pancreatic β cells injury and reduced FBG level in T1DM rats.**

Rats were divided into six groups: blank controls (Control); T1DM rats (Model); T1DM rats treated with metformin (MET); T1DM rats treated with low-dose[25mg/kg(bw)/day] UA(UA-L), middle-dose[50mg/kg(bw)/day] UA(UA-M) and high-dose[100mg/kg(bw)/day] UA(UA-H). (A) Body weight change: each group of rats was weighted every week during the observation period (n=10-12). (B) Fasting blood glucose levels (n=10-12). (C) Histologic changes of the pancreatic islets and the insulin secretion (shown by black arrows) were detected by IHC staining, original magnification ×400 (scale bar, 200 μm). Data are expressed as the mean ± SD. ***P*<0.01 versus the control, *P<0.05 versus the control; ^##^*P*<0.01 versus Model，^#^*P*<0.05 versus Model.

**3.2 UA treatment altered the specific microbiota population in T1DM rats**

To investigate the role of the gut microbiota in T1DM and the effect of UA, we analyzed the relative abundances, diversity and structure of gut microbiota in stool samples using 16S rDNA sequencing. A total of 1231141 high-quality reads from 24 samples were generated. Based on 97% similarity, 817 OTUs were obtained, which were divided into 12 phyla, 19 classes, 32 orders, 62 families, and 166 genera. As can be seen from the Pan/Core curve (Fig 2A), as the sample size of each group increased to 4, the increase in the total OTU number and the decrease of the total OTU number of each group tended to be gentle, indicating that the detection amount of each group was sufficient.

The results of alpha diversity analysis, reflecting the diversity and abundance of colony species, are shown in (Fig 2B). Our results showed that the Shannon and Simpson values were significantly different among the control, model, MET, and UA-M groups suggesting that MET and UA can increase the diversity of the [gut microbiota](https://www.sciencedirect.com/topics/pharmacology-toxicology-and-pharmaceutical-science/intestine-flora) in T1DM rats. However, there were no significant differences in Chao and Sobs values among the groups. Therefore, the abundance of [gut microbiota](https://www.sciencedirect.com/topics/pharmacology-toxicology-and-pharmaceutical-science/intestine-flora)  in each group was not affected. Principal co-ordinates analysis (PCoA) of the unweighted Unifrac distances was used to evaluate the differences in diversity of the intestinal microbiota among the samples and groups (Fig 2C). The result revealed that the distribution of species in the Model and UA-L groups was separate from that in the control group, whereas the MET, UA-M, and UA-H groups were closer to the control group.

Next, we performed taxonomy-based analysis at the phylum and genus levels to evaluate the community composition of the gut microbes of rats in each group. The results showed that the composition of the gut microbiota in both the UA-M and control groups was similar. The results also indicated that the gut microbiota includes five major phyla: *Firmicutes, Bacteroidetes, Actinobacteria, Verrucomicrobia, and Proteobacteria*. Among them, *Firmicutes* and *Bacteroidetes* were the dominant flora in each group, and their sum accounted for more than 70%. Compared to control rats, there was a reduction in *Firmicutes* and an increase in *Bacteroidetes* in model rats, but UA remarkably modulated the levels of *Firmicutes* and *Bacteroidetes* (Fig 2D-E). At the genus level, we found that the abundances of bacterial genera was significantly different among the six groups. The predominant bacterial genera in the UA and control groups were *Lactobacillus* and *Romboutsia*, while the abundances of *Lactobacillus* and *Romboutsia* in the model group declined. Meanwhile, *norank_f_Muribaculaceae* was the predominant bacterial genus in the model group, while the abundances of *norank_f_Muribaculaceae* was decreased in the UA and control groups (Fig 2F-G). Linear [discriminant analysis](https://www.sciencedirect.com/topics/medicine-and-dentistry/discriminant-analysis) (LDA) coupled with effect size measurements (LEfSe) was used to further identify the specific bacterial taxa that significantly differed in response to UA treatment. According to the analysis results, *Bacteroidetes*, *Verrucomicrobia* and *Proteobacteria* were the key types of bacteria contributing to gut microbiota dysbiosis in the Model group. Nevertheless, *Firmicutes*, *Actinobacteria*, *Bifidobacteriales,* and *Lactobacillales* displayed relative enrichment in the UA and MET groups, which might be associated with the UA-mediated and MET- mediated alleviation of T1DM. (Fig 2H-I). The results demonstrated that T1DM led to a gut microbiota disorder, and UA treatment could alter the gut microbiota diversity and composition.

**Fig 2.UA treatment altered the specific microbiota population in T1DM rats.**

To assess the effects of UA on gut microbiota, 16S rRNA sequencing of rat fecal DNA was performed. (A) Pan/core species analysis. The Pan/Core curve showed that the detection amount of each group is sufficient. (B) The alpha diversity analysis reflected the diversity and abundance of gut microbiota. (Chao index, Sobs index, Shannon index, and Simpson index). (C) Unweighted UniFrac-based principal coordinates analysis (PCoA) at the OUT level indicated the different β-diversity of gut microbiota. The ANOSIM analysis of variance confirmed the statistically significant separation of the six groups (R=0.821, *P*＜0.05). (D) The relative abundance of different bacterial phyla in each group. (E) A phylum-level differential analysis of these six groups. (F) Relative abundance of different bacterial genera in each group. (G) The differential analysis among these six groups at the genus level. (H) Linear discriminant analysis effect size (LEfSe) taxonomic cladogram depicting taxonomic association between microbiome communities from six groups. Each node represents a specific taxonomic type. Yellow nodes denote the taxonomic features that are not significantly differentiated in each group. (I) Linear discriminant analysis (LDA) score derived from differentially abundant features in six groups. The LDA threshold was set to 2, and *P*<0.05 when LDA>2. (A-I) n = 4. Data are expressed as the mean ± SD. ***P*<0.01 versus the control, **P*<0.05 versus the control; ^##^*P*<0.01 versus Model，^#^*P*<0.05 versus Model.

**3.3UA treatment corrected the proportion of Th17 and Treg cells in rat with T1DM**

To investigate the potential mechanism of UA in correcting Th17/Treg differentiation in T1DM rats, we assessed the differentiation of Th17/Treg cells before and after UA intervention. As shown in Fig 3A-B, UA-treated rats had a significantly lower numbers of Th17 cells (Fig 3C), and a higher number of Treg cells than that in T1DM rats (Fig 3D). In addition, the proportion of Th17 and Treg cells in T1DM rats changed, and UA treatment alleviated this trend (Fig 3E).

To confirm our flow cytometry results, we measured serum levels of cytokines such as IL-17A, IL-10 and TNF-α (Fig 3F-H), as well as the mRNA and protein expression of Th17/Treg-associated genes such as RORγt and Foxp3in T1DM rats (Fig 3I-M). These results revealed that the levels of Foxp3 and IL-10 in control and UA rats were significantly higher than those in model rats, while the expression of RORγt, IL-17A, and TNF-α in UA rats was significantly lower than that in model rats. These results suggest that UA can improve T1DM symptoms by correcting the imbalance in Th17/Treg cell differentiation and regulating the secretion of inflammatory factors.

**Fig 3.UA treatment corrected the proportion of Th17 and Treg cells in rat with T1DM.**

(A) CD4^+^IL-17A^+^ (Th17) cells and (B) CD4^+^CD25^+^Foxp3^+^ (Treg) cells in the spleen from six groups were analyzed by flow cytometry. (C) The Th17/Treg ratio in the spleens. (D)The percentage of CD4^+^IL-17A^+^(Th17) cells in the spleens. (E) The percentage of CD4^+^CD25^+^Foxp3^+^ (Treg)cells in the spleens. (n=6). ELISA was used to compare the levels of cytokines IL-17A (F), IL-10 (G), and TNF- (H) in blood serum between six groups (n = 8). mRNA expression levels of RORγt (I) and Foxp3 (J) in the spleen of the rats were measured by real-time quantitative PCR (n=6). The relative expression of RORγt (K) and Foxp3 (L) proteins. (M)Western blotting results of RORγt, Foxp3, and β-actin in the spleen of T1DM rats(n=3). The density of protein bands was quantified by software Alpha, which was normalized by comparison with β-actin and expressed as a percentage of the control. The data are expressed as the mean ± SD. ***P*<0.01 versus the control, **P*<0.05 versus the control; ^##^*P*<0.01 versus Model，^#^*P*<0.05 versus Model.

**3.4 Associations between gut microbiota composition and T1DM phenotypes**

RDA analysis and correlation heat-map analysis were applied to investigate the correlations between the relative abundance of the different gut microbial communities and T1DM phenotypes (body weight, FBG, Th17 cells, Treg cells, IL-10, IL-17A, TNF-α, RORγt, and Foxp3). The results from the RDA analysis showed that Treg cells were most closely correlated with the distribution of the sample gut microbes (Fig 4A).

At the phylum level, we found that *Patescibacteria* and *Deferribacteres* were negatively correlated with Th17 cells and the Th17/Treg ratio, but positively correlated with Treg cells. However, both *Verrucomicrobia* and *Cyanobacteria* exhibited a positive correlation with Th17 cells and the ratio of Th17/Treg (Fig 4B). At the genus level, *Lactobacillus* showed positive correlations with Treg cells, IL-10, and Foxp3 but were negatively correlated with IL-17A and RORγt. Seven genera including *Phascolarctobacterium* exhibited a positive correlation with IL-17A, Th17cells, the ratio of Th17/Treg and RORγt, and a negative correlation with Treg cells and Foxp3. Six genera were positively associated with FBG but negatively associated with body weight (Fig 4C).

**Fig 4.Associations between gut microbiota composition and T1DM phenotypes.**

*2. Figure 2F is very wordy and the text is unclear even if the large image is downloaded. Please increase clarity of the figure.*

**Reply:** Thank you for pointing this out. Due we have relabeled all the figures to match the figure file name and the caption label with the corresponding in-text citations in our manuscript. And Fig 2F in the original manuscript has been replaced with Fig 2H-I. The Fig 2H-I.tif as clear as possible have been provided.

**Fig 2H.** Linear discriminant analysis effect size (LEfSe) taxonomic cladogram depicting taxonomic association between microbiome communities from six groups. Each node represents a specific taxonomic type. Yellow nodes denote the taxonomic features that are not significantly differentiated in each group. Control: blank controls; Model: T1DM rats; MET: T1DM rats treated with metformin; UA-L: T1DM rats treated with low-dose UA; UA-M: T1DM rats treated with middle-dose UA; UA-H: T1DM rats treated with high-dose UA.

**Fig 2I.** Linear discriminant analysis (LDA) score derived from differentially abundant features in six groups. The LDA score was calculated on the horizontal coordinate, and the species name was calculated on the vertical coordinate. The LDA discrimination bar graph counted the microbial groups with significant effects in all groups, and the LDA score was obtained by LDA analysis. The LDA threshold was set to 2, and P<0.05 when LDA>2. Control: blank controls; Model: T1DM rats; MET: T1DM rats treated with metformin; UA-L: T1DM rats treated with low-dose UA; UA-M: T1DM rats treated with middle-dose UA; UA-H: T1DM rats treated with high-dose UA.

*3. The change in immune cells seems to be occurring in the spleen. Have the authors also looked at local tissue? Peyer’s patches, mesenteric lymph nodes and lamina propria? If not, could you speculate how gut microbiota can impact splenic Th17/Treg differentiation? What is the effect of UA?*

**Reply:** We greatly appreciate for your thoughtful comments. Our group has also studied changes in immune cells in local tissues such as mesenteric lymph nodes. The result revealed that the model group had a significantly higher numbers of Th17 cells (*P*<0.01), and a lower of Treg cells (*P*<0.01) than that in control group after six weeks treatment. Besides, the ratio of Th17 and Treg cells increased significantly in model group. Compare to model rats, there was a reduction in Th17 cells and an increase in Treg cells in the UA group (S1 Fig). The finding is consistent with the results of this studies.

**S1 Fig: UA treatment corrected the proportion of Th17 and Treg cells in rat with T1DM.**

(A) CD4+IL-17A+ (Th17) cells and (B) CD4+CD25+Foxp3+ (Treg) cells in the mesenteric lymph nodes from six groups were analyzed by flow cytometry. (C) The percentage of CD4+IL-17A+(Th17) cells in the mesenteric lymph nodes. (D) The percentage of CD4+CD25+Foxp3+ (Treg)cells in the mesenteric lymph nodes. (E) The Th17/Treg ratio in the mesenteric lymph nodes. (n=6). The data are expressed as the mean ± SD. **P<0.01 versus the control, *P<0.05 versus the control; ##P<0.01 versus Model，#P<0.05 versus Model.

We hypothesized that gut microbiota mainly affects Th17/Treg cell differentiation from the following three aspects: Firstly, Gut microbiota may affect Th17/Treg balance through energy metabolism pathway. It is well accepted that commensal bacteria prime Th17 differentiation. In particular, adenosine 5′-triphosphate (ATP) derived from commensal bacteria has been shown to activate CD11^clow^CD70^high^ cells in the lamina propria, driving Th17 cells differentiation ^[1]^. Lactobacillus can also up-regulate genes related to carbohydrate transport and metabolism. When carbohydrate utilization increases, so does the amount of short-chain fatty acids (SCFAs) involved in lipid and glucose metabolism ^[2]^. And the SCFAs participates in the generation of Tregs by suppressing proinflammatory cytokines, by promoting RA production from DCs, and by inducing Foxp3 transcription. LEfSe analysis in our study showed that UA mainly changes the composition of the gut microbiota by enriching Bifidobacterium and Lactobacillus. Therefore, we hypothesized that UA may enhance the expression of SCFA by enriching Lactobacillus, and then promote the differentiation of Treg cells. Secondly, the gut microbiota can directly affect the Th17/Treg differentiation direction through genes. *Bacteroidetes* can regulate the activity of antimicrobial peptides through Toll-like receptors in intestinal mucosal cells, thus intervening in the innate immune system and the differentiation of naive T cells. Compared to control rats, there was a reduction in *Bacteroidetes* in model rats, and UA remarkably modulated the levels of *Bacteroidetes.* Therefore, UA may also affect the enrichment of *Bacteroidetes* by regulating them. Thirdly, the gut microbiota plays a role in Th17/Treg immune balance through cytokines. Polysaccharide A, the most abundant capsular polysaccharide expressed by *B. fragilis*, mediates conversion of CD4+ T-cells into IL-10-producing Foxp3+ Tregs via TLR2 and suppresses Th17 responses ^[3]^. Segmented filamentous bacteria can induce Th17 differentiation by promoting IL-17 expression in RORγT + T cells ^[4]^. Our study found that UA treatment could reduce the level of TL-17, increase the expression of IL-10, and regulate the abundance and diversity of gut microbiota in T1DM rats. Therefore, we hypothesized that UA could regulate cytokines and affect the differentiation of Th17/Treg, which may be related to gut microbiota.

**Reference：**

1.Atarashi K., Nishimura J., Shima T., et al. ATP drives lamina propria TH17 cell differentiation. *Nature*. 2008;455(7214):808–812.

2. Carding S,Verbeke K,Vipond DT,et al. Dysbiosis of the gut microbiota in disease[J]. Microb Ecol Health Dis,2015,26(112):26191.

3. Round JL, Lee SM, Li J, Tran G, Jabri B, Chatila TA, et al. The toll-like receptor 2 pathway establishes colonization by a commensal of the human microbiota. Science (2011) 332:974–7.

4. Atarashi K, Tanoue T, Ando M, Kamada N, Nagano Y, Narushima S, et al. Th17 cell induction by adhesion of microbes to intestinal epithelial cells. Cell (2015) 163:367–80.

*4. All the beneficial effects of UA were similar to that of metformin an already well tolerated drug. Are there any side effects of UA? What is the benefit of using it over metformin? Please discuss*

**Reply:** Thanks for your professional comments. The benefit of using UA over metformin in our study is stated clearly in discussion section in Page 15-16 line 341-360 as shown below:

T1DM is characterized by the immune-mediated depletion of β-cells, resultanting in insulin deficiency and hyperglycaemia. Metformin could decrease glucose levels [28-30] and improves insulin sensitivity [31]. The American Diabetes Association has also made a positive recommendation for adding metformin to insulin therapy in overweight patients with T1DM [32]. Therefore, metformin was used as a positive control in our study.

UA is a natural pentacyclic triterpenoid derived from fruit and various traditional Chinese medicinal plants. It also possesses important antidiabetic effects, including lowering blood glucose levels and preserving pancreatic islet cells function [27, 33, 34]. For instance, UA exerts hypoglycemic effects by inhibiting the activities of α-amylase and α-glucosidase [35]; activating TGR5 to enhance GLP-1secretion in type 1-like diabetic rats [36]; and reducing the activity of protein tyrosine phosphatase 1 B [37].Compared to metformin, UA has been shown to exhibit poor oral bioavailability, low solubility, and intestinal permeability [38]. However, metformin is associated with rare but serious events of lactic acidosis and gastrointestinal sideeffects [39, 40], yet UA did not exhibit any significant toxicity in human THP-1 cell [41]. Moreover, a long-term toxicity study showed that oral dosing of UA for 90 consecutive days did not lead to toxic effects at any of the doses. In contrast, recent studies have reported that gut microbiota dysbiosis and the differentiation of Th17/Treg cells contribute to T1DM progression [11, 17, 42]. In addition, UA modulates the abundance and diversity of intestinal flora and inhibit Th17 cells differentiation [43, 44]. However, whether UA has similar protective and ameliorative effects on T1DM remains unknown.

*5. Please state molecular weight of proteins on the representative western blots*
**Reply:** Thank you for your significant reminding. The molecular weight of proteins on the western blots has been stated.

**Minor concerns**
*1. The authors have mentioned that a recent study has shown there is an imbalance in Th17 and Tregs (this has been published in 2019) Is there any other recent study showing this?*

**Reply:** We gratefully appreciate for your comment. There are some other recent studies have reported similar results. Starosz A and his colleagues assessed the changes within Treg and Th17 cells in T1DM pediatric patients and their association with disease remission, their results revealed that Treg and ratio Treg/Th17 are significantly higher in T1D than in healthy controls (published in 2022). Chen *et al*. found that Anti-CD20 therapy might have some beneficial effects on T1DM through regulation of Th17/Treg cells and the proinflammatory/anti-inflammatory balance (published in 2022).

**Reference**

1.Starosz A, Jamiołkowska-Sztabkowska M, Głowińska-Olszewska B, Moniuszko M, Bossowski A, Grubczak K. Immunological balance between Treg and Th17 lymphocytes as a key element of type 1 diabetes progression in children. Frontiers in immunology. 2022; 13:958430.

2. Chen M, Zhang Q, Wei Y, Wan Q, Xu M, Chen X. Anti-CD20 therapy ameliorates β cell function and rebalances Th17/Treg cells in NOD mice. Endocrine. 2022;76(1):44-52.

*2. Introduction is initiated with an abbreviation, please avoid doing so.*

**Reply:** We are very sorry for our careless mistake and Thank you for reminding us this important point. According to your suggestion, the full descriptions of the abbreviations like T1DM, NOD etc have been supplemented in the revised manuscript in page 2 line 33-34 and page 3 line 59-62 as following:

**page 2 line 33-34:** Type 1 diabetes mellitus (T1DM) is a chronic metabolic disease in which the autoimmune system progressively damages the pancreatic β-cells, leading to insulin deficiency.

**page 3 line 59-62:** In addition, Kim et al. reported that the microbial combination could be effective in autoimmune diabetes in non-obese diabetic (NOD) mice by altering gut permeability, increasing the generation of gut-homing Treg cells and reduced Th1 polarization.

*3. Scientific names should be in italics Page 4 line 90-91 (Salvia officinalis etc.) Please correct.*

**Reply:** Thank you for your valuable suggestion. We have italicized the scientific names and made a comprehensive check on the full manuscript, and the modification are as follows:

**page 4 line 79-81:** Ursolic acid (UA, 3β-hydroxyurs-12-en-28-OIC acid) is a pentacyclic triterpenoidderived from medicinal plants such as *Salvia officinalis, Lavandula angustifolia, Melissa officinalis, Ocimum basilicum, Origanum majorana and Satureja montana.*

**page 3 line 50-52:** A case-control study suggested that the children with T1DM have a significant increase in the quantity of *Bacteroidetes* and a significant decrease in the number of *Firmicutes* and *Actinobacteria*, and they also have a lower *Firmicutes* to *Bacteroidetes* ratio.

**Page 17 line 371-374:** By comparing the gut microbiota of T1DM Han Chinese patients and those of healthy subjects, Huang et al. found that the gut microbiota of T1DM patients was significantly different, with the ratio of *Bacteroides/Firmicutes* increasing significantly.

**Answer Reviewer #2:**

*1.In this manuscript, the authors describe their studies on the effects of ursolic acid (UA) in reversing gut microbiota dysbiosis and imbalanced Th17/Treg cells in T1DM rats. The experiments are technically well-designed and study results are appropriately described. However, rationale for choosing UA (in preference to many other phytochemicals showing hypoglycemic, antioxidant, anti-inflammatory, antitumor and neuroprotective properties) is not well addressed.*

**Reply:** We thank you for your careful reading of our manuscript and providing us with these keen scientific insights. We provide a detailed explanation of the rationale for choosing UA.

Ursolic acid (UA, 3β-hydroxyurs-12-en-28-OIC acid) is a pentacyclic triterpenoidderived from medicinal plants such as *Salvia officinalis, Lavandula angustifolia, Melissa officinalis, Ocimum basilicum, Origanum majorana and Satureja montana* [20]. It has various pharmacological activities, such as hypoglycemic, antioxidant, anti-inflammatory, antitumor and neuroprotective [21-23]. UA exhibits anti-cancer effects by inducing tumor cell cycle arrest and apoptosis. It also suppresses transformation and inhibits tumor angiogenesis and tumorsphere formation. Morever, the neuroprotective effects of UA could be associated with its ability to stimulate cellular antioxidant defense systems and downregulate the pro-inflammatory pathways in neurodegeneration models *in vitro* and *in vivo* [20, 24, 25]. The hypoglycemic effect of ursolic acid is generally recognized. Tang *et al*. [26] found that UA could decrease oxidative stress in pancreatic tissue to ensure the regeneration of pancreatic β-cells and, therefore, pancreatic insulin. It has also been suggested that UA exhibits potential anti-diabetic effects, as it protects islet cells from streptozotocin (STZ)-induced damage and modulates blood glucose levels [27]. Despite the well-documented excellent hypoglycemic activity of UA, little is known about its potential pharmacological effects on T1DM, Th17/Treg cell balance and gut microbiota.

Therefore, in this study we examined whether UA exerts protective effects in rat with STZ-induced T1DM. We also investigated the effect of UA on gut microbial composition and Th17/Treg balance.

*2.There are multiple grammatical or typo errors that need attention (for example type I diabetes, not diabetic, in the Abstract).*

**Reply:** We are very sorry for our careless mistake and Thank you for reminding us this important point. All article has been edited by professionals, and the edit certificate has been uploaded. Revised portion is marked in red on the paper to mark all changes clearly. A screenshot of the retouching certificate as follows:

*3.Also, the statements like “The results probably provided new research strategies for the prevention and treatment of T1DM” significantly dampens the scientific merit of the study.*

**Reply:** Thank you for your careful guidance. Your suggestions are conducive to better reflect the scientific value of our research, which will have a good guiding effect on our future scientific research work. In the revised version of the manuscript, we have adjusted the similar statement in abstract and discussion.

**page 2 line 29-31:** These findings suggest that UA can lower FBG levels in T1DM rats, delay the progressive destruction of pancreatic β-cells, and modulate gut microbiota homeostasis and immune function in rat with streptozotocin-induced T1DM.

**page 19 line 433-438:** Our study demonstrated that UA alleviates the symptoms of STZ-induced T1DM in rat. UA treatment reduced FBG levels, delayed the progressive destruction of pancreatic β-cells, modulated intestinal microflora composition, and restored Treg/Th17 cell balance. Collectively, these results suggest that UA is a promising drug for the prevention and treatment of T1DM. However, further research is required to confirm the positive results of this study.

*4. Further, the studies described here are far from confirmatory to infer whether the ameliorating effects of UA on T1DM complications are direct effects of UA or mediated via correcting dysbiosis. This can be confirmed only by direct treatment with UA in vitro in cell culture experiments or in vivo in germ free animals. Overall, the manuscript needs major revision with additional studies to confirm the claim that UA effects are via gut microbiota, not direct effects, or by downplaying the claim by revised writing of the Abstract, Results and Discussion/Conclusion sections.*

**Reply:** Thanks for your professional comments. Indeed, our study will be more profound if we confirm the claim that UA effects are via gut microbiota, not direct effects. We would to use germ free rats to carry out related research in our next experiment. AS the results of experimental cannot be obtained in a short period of time, they cannot be shown in this article. Therefore, we seek for your tolerance and understanding. And we have revised the manuscript to downplay this claim by revising the writing of the Abstract, Results and Discussion/Conclusion sections as you suggested. Specific modifications are as follows:

**Abstract**

Ursolic acid (UA), a natural pentacyclic triterpenoid obtained from fruit and several traditional Chinese medicinal plants, exhibits anti-inflammatory and hypoglycemic properties. However, its protective effects against type 1 diabetes mellitus (T1DM) are being explored. In this study, streptozotocin-induced T1DM rat models were established and treated with UA for six weeks. T1DM rats treated with UA were used to observe the effects of UA on body weight and fasting blood glucose (FBG) levels. Pathological changes in the pancreas were observed by immunohistochemical staining. The gut microbiota distribution was measured using 16S rDNA high-throughput sequencing. The proportion of Th17 and Treg cells were examined by flow cytometry. Protein and mRNA expression of molecules involved in Th17/Treg cell differentiation were assessed by quantitative real-time PCR and western blotting. The correlation between gut microbiota and Th17/Treg cell differentiation in T1DM was analyzed using redundancy analysis (RDA) analysis. Compared to the model group, FBG levels declined, and the progressive destruction of pancreatic β cells were alleviated. The diversity and uniformity of the gut microbiota in T1DM rats treated with UA increased significantly. Interestingly, the Th17/Treg cell differentiation imbalance was corrected and positively correlated with the expression of Foxp3 and IL-10 and negatively correlated with the expression of RORγt, IL-17A, and TNF-α. These findings suggest that UA can lower FBG levels in T1DM rats, delay the progressive destruction of pancreatic β-cells, and modulate gut microbiota homeostasis and immune function in rat with streptozotocin-induced T1DM.

**Results**

**3.1UA treatment alleviated pancreatic β cells injury and reduced FBG level in T1DM rats.**

Notably, histological analysis showed that the model group displayed distinctly abnormal islet structures compared with those of the control group; they appeared as small islets with irregular morphology and indistinct boundaries with surrounding tissue, inhomogeneous islet cells, cytoplasmic vacuolation, fat vacuoles, and infiltration of inflammatory cells into islets. However, UA-treated rats had a significantly larger islet surface area and a lower number of insulin-positive cells in the islets compared to the rats in the model group (Fig 1C). Together, these results indicated that UA treatment significantly ameliorated STZ-induced T1DM.

**3.2 UA treatment altered the specific microbiota population in T1DM rats**

Principal co-ordinates analysis (PCoA) of the unweighted Unifrac distances was used to evaluate the differences in diversity of the intestinal microbiota among the samples and groups (Fig 2C). The result revealed that the distribution of species in the Model and UA-L groups was separate from that in the control group, whereas the MET, UA-M, and UA-H groups were closer to the control group.

Next, we performed taxonomy-based analysis at the phylum and genus levels to evaluate the community composition of the gut microbes of rats in each group. The results showed that the composition of the gut microbiota in both the UA-M and control groups was similar. The results also indicated that the gut microbiota includes five major phyla: *Firmicutes, Bacteroidetes, Actinobacteria, Verrucomicrobia, and Proteobacteria*. Among them, *Firmicutes* and *Bacteroidetes* were the dominant flora in each group, and their sum accounted for more than 70%. Compared to control rats, there was a reduction in *Firmicutes* and an increase in *Bacteroidetes* in model rats, but UA remarkably modulated the levels of *Firmicutes* and *Bacteroidetes* (Fig 2D-E). At the genus level, we found that the abundances of bacterial genera was significantly different among the six groups. The predominant bacterial genera in the UA and control groups were *Lactobacillus* and *Romboutsia*, while the abundances of *Lactobacillus* and *Romboutsia* in the model group declined. Meanwhile, *norank_f_Muribaculaceae* was the predominant bacterial genus in the model group, while the abundances of *norank_f_Muribaculaceae* was decreased in the UA and control groups (Fig 2F-G).Linear [discriminant analysis](https://www.sciencedirect.com/topics/medicine-and-dentistry/discriminant-analysis) (LDA) coupled with effect size measurements (LEfSe) was used to further identify the specific bacterial taxa that significantly differed in response to UA treatment. According to the analysis results, *Bacteroidetes*, *Verrucomicrobia* and *Proteobacteria* were the key types of bacteria contributing to gut microbiota dysbiosis in the Model group. Nevertheless, *Firmicutes*, *Actinobacteria*, *Bifidobacteriales,* and *Lactobacillales* displayed relative enrichment in the UA and MET groups, which might be associated with the UA-mediated and MET- mediated alleviation of T1DM. (Fig 2H-I). The results demonstrated that T1DM led to a gut microbiota disorder, and UA treatment could alter the gut microbiota diversity and composition.

**3.3UA treatment corrected the proportion of Th17 and Treg cells in rat with T1DM**

These results suggest that UA can correct the imbalance in Th17/Treg cell differentiation and regulating the secretion of inflammatory factors.

**3.4** **Associations between gut microbiota composition and T1DM phenotypes**

**Discussion**

In our study, we established a STZ-induced rat model of T1DM and found that UA can improve disease symptoms and pathological characteristics. In addition, our results preliminarily demonstrated that UA could increase the diversity of the gut microbiota and correct the imbalance of Th17/Treg cells in T1DM rats.

Increasing evidence indicates that the gut microbiota is involved in the occurrence and development of T1DM[45], and gut microbiota dysbiosis exists in diabetic patients. Fassatoui *et al*. studied the fecal bacterial composition of 31 participants, and the results showed that the proportions of *Firmicutes* and the ratio *Firmicutes/Bacteroidetes* decreased in participants with T1DM compared to those without diabetes [11]. Moreover, a study revealed that the gut microbiota of newly diagnosed T1D cases was altered, with higher levels of the genus *Bacteroides* compared to those of the control group [46]. By comparing the gut microbiota of T1DM Han Chinese patients and those of healthy subjects, Huang *et al.* found that the gut microbiota of T1DM patients was significantly different, with the ratio of *Bacteroides/Firmicutes* increasing significantly [47]. Our study showed that the diversity and abundance of gut bacteria in T1DM rats were lower than those in controls. Beta diversity analysis showed a reduction in *Firmicutes* and the ratio of *Firmicutes/Bacteroidetes* but an increase in *Bacteroidetes*, *Actinobacteria*, *Verrucomicrobia* and *Proteobacteria* in model rats. The above results also confirmed that there were certain similarities between the model rats used in our study and T1DM patients. Interestingly, one study [48] demonstrated that specific inulin-type fructan fibers could delay the development of T1DM by modulating microbiota homeostasis. Zhao *et al.* reported that cinnamaldehyde may interfere with gut microbiota to modulate the expression levels of glucose metabolism-related genes, thus subsequently reducing blood glucose levels in T1DM mice [49]. In this study, we demonstrated for the first time that UA can increase the diversity and abundance of gut bacteria in T1DM rats. Specifically, UA treatment significantly increased the abundance of *Firmicutes* and the ratio of *Firmicutes* to *Bacteroidetes*, and significantly decreased the abundance of *Bacteroidetes*, *Actinobacteria*, *Verrucomicrobia* and *Proteobacteria*. LEfSe analysis showed that UA mainly changes the composition of the gut microbiota by enriching *Bifidobacterium* and *Lactobacillus*. These findings suggest that UA may play an important role in restoring [homeostasis](https://www.sciencedirect.com/topics/medicine-and-dentistry/homeostasis) of gut microbiota microecology in T1DM.

Among CD4^+^ cells, Th17 cells cause autoimmunity and inflammation, whereas Treg cells maintain immune tolerance to self-antigen and inhibit inflammatory immune response [50]. They are considered the main effector subgroups involved in the pathogenesis of T1DM. Treg cells have been shown to be beneficial in the treatment of T1DM [51], and inhibiting Th17 cells differentiation may significantly delay the development of T1DM [52]. Ryba-Stanisławowska *et al.* found that the Treg/Th17 balance was disrupted in patients with T1DM, which may hasten the development of [diabetic complications](https://www.sciencedirect.com/topics/medicine-and-dentistry/complications-of-diabetes-mellitus)[53]. Ferraro *et al*. demonstrated that there was a Th17-cell bias and/or a Treg cell defect in patients with T1DM; thus, treatments targeted at suppressing Th17 cells and/or refitting Tregs may represent a solution for diabetic patients [54]. Moreover, the differentiation of Th17 and Treg cells is directed by the master transcription factor RORγt and Foxp3, respectively. Th17 cells secrete the pro-inflammatory cytokine IL-17, which directly damage pancreatic β cells [17]. TNF-α is involved in the pathogenesis of T1DM by destroying β-cell function, inhibiting insulin secretion, and inducing apoptosis[55]. In contrast, IL-10 an anti-inflammatory cytokine produced by Treg cells [56], inhibits the progression of diabetes in an animal model [57]. Our study showed that there was a significantly lower numbers of Treg cells and IL-10, but a higher number of Th17 cells, IL-17A, and TNF-α in T1DM rats than controls. This finding is consistent with the results of previous studies. Furthermore, Duan *et al*. found that metformin treatment significantly mitigated autoimmunity in NOD mice by inhibiting the differentiation of Th17 cells while promoting the development of Tregs [18]. Xu *et al*. reported that UA ameliorated the symptoms of experimental autoimmune myasthenia gravis by prompting Th17 cells to produce Th2 cytokines and up-regulate Treg cells [58]. Gudi *et al*. demonstrated that oral administration of high‐purity yeast β‐glucan suppressed insulitis by increasing the expression of IL10 and Foxp3+ T‐cell frequencies in NOD mice [59]. Our study confirmed that UA dramatically inhibited Th17 cells, raised Treg cells, and improved the Th17/Treg balance in T1DM. Moreover, Th17 cells markers such as RORγt, IL-17A and TNF-αdecreased, and Treg markers such as FoxP3 and IL-10 increased. Our data indicated that UA could not only correct the imbalance of Th17/Treg cells but also affect the differentiation of its cytokines to inhibit the inflammatory response, thus delaying the progression of T1DM.

The gut microbiome primarily influences the progression of immune diseases by regulating the Th17/Treg axis[60]. *Lactobacilli* and *Bifidobacteria* have been reported to be beneficial microbes, because they inhibit Th17 cell differentiation and increased Treg cell differentiation [61]. Th17 cells are produced by the colonization of *Bacteroides fragilis* [62], while Treg cell accumulation can also be induced by *Clostridia* [63]. Chen *et al*. found that both *C. butyricum* and norfloxacin treatment reduced Th17 cells, and increased the percentages of Tregs, and the gut microbiota alteration may be responsible for this effect [64]. Our study showed that UA treatment increased the levels of *Lactobacillus*, *Bifidobacterium* and *Ruminococcaceae_UCG-014*, but decreased the levels of *Bacteroidetes*. This indicates that UA modulates the abundance of gut microbiota related to the differentiation of Th17 and Treg cells. However, the mechanism by which the gut microbiota modulate the differentiation of Th17/Treg during UA treatment is unclear and requires further investigation.

**Conclusions**

Our study demonstrated that UA alleviates the symptoms of STZ-induced T1DM in rat. UA treatment reduced FBG levels, delayed the progressive destruction of pancreatic β-cells, modulated intestinal microflora composition, and restored Treg/Th17 cell balance. Collectively, these results suggest that UA is a promising drug for the prevention and treatment of T1DM. However, further research is required to confirm the positive results of this study.

In all, we found the editor and reviewer’s comments are quite helpful, and we tired our best to improve the manuscript and made some changes in the manuscript. Finally, we would like to express our great appreciation to all comments on our paper. We hope that the manuscript will be accepted for publication in PLOS ONE.

The manuscript has been resubmitted to the system. We look forward to your positive response. Thank you and best regards.

Yours sincerely,

Xiaoli Zhang
